# Synthetic Expansion of Blood Dielectric Spectra at Microwave Frequencies Using Data-Driven Methods

**DOI:** 10.3390/s26113580

**Published:** 2026-06-04

**Authors:** Iman Alhummada, Alina Bialkowski, Lei Guo, Wilbert Villena Gonzales, Mohamed Deriche, Amin Abbosh

**Affiliations:** 1 School of Electrical Engineering and Computer Science, University of Queensland, Brisbane, QLD 4072, Australia; alina.bialkowski@uq.edu.au (A.B.); l.guo3@uq.edu.au (L.G.); w.villena@uq.edu.au (W.V.G.); a.abbosh@uq.edu.au (A.A.); 2 College of Engineering and Information Technology, AIRC, Ajman University, Ajman P.O. Box 346, United Arab Emirates; m.deriche@ajman.ac.ae

**Keywords:** synthetic data, hemoglobin, Gaussian Process Regression, Earth Mover’s Distance, Cole–Cole model, XGBoost

## Abstract

Accurate characterisation of blood dielectric properties is essential for data-driven biomedical sensing, yet experimental datasets are often limited to a few discrete hemoglobin (Hb) concentrations. This constraint hinders the development of robust data-driven models. To address this, the present study introduces a framework for generating synthetic blood permittivity spectra from sparse measurements. Four data-generation strategies were investigated, combining interpolation-based techniques and probabilistic models to extend Hb-dependent spectral coverage across the measured frequency range. Model performance was evaluated using Earth Mover’s Distance (EMD) for spectral similarity, Cole–Cole parameter analysis for physical consistency, variance preservation metrics, and Hb prediction using XGBoost. The results indicate that interpolation-based approaches achieve the highest reconstruction accuracy, while Conditional Bayesian principal component analysis (Conditional BPCA) produces smooth and physically consistent spectra with stable variability characteristics. Across all methods, the generated datasets maintained sufficient Hb-related information to support reliable prediction. These findings demonstrate that the proposed framework enables effective expansion of limited dielectric datasets while supporting a multi-criteria evaluation of synthetic data quality, including fidelity, variability, and predictive relevance.

## 1. Introduction

Microwave sensing is increasingly investigated for medical diagnostics due to its sensitivity to the dielectric properties of biological tissues, which vary with physiological and pathological conditions [1]. Dielectric properties are key parameters that govern how externally applied electromagnetic fields interact with the human body and how these fields are distributed within biological tissues [2]. In particular, they affect wave propagation, scattering, and absorption, forming the basis of microwave sensing and imaging techniques [3]. In blood, dielectric permittivity varies systematically with physiological state, producing electromagnetic signatures that reflect changes in hematocrit (Hct), defined as the volume fraction of red blood cells (RBCs) in blood [4], as well as changes in hemoglobin (Hb) levels, which are related to the underlying RBC mass [5]. This relationship supports the use of permittivity for Hb estimation and anemia detection, as measurable shifts in the dielectric response reflect underlying changes in blood composition [6]. As microwave sensing-based diagnostic approaches continue to advance, this behavior provides a credible basis for non-invasive sensing and continuous physiological assessment. The feasibility of this approach is supported by extensive dielectric characterization studies showing that tissue composition produces distinct electromagnetic responses across frequency [7].

Despite these advantages, acquiring blood permittivity measurements over a broad and densely sampled range of Hb levels remains inherently challenging. More generally, dielectric characterization of biological tissues is experimentally demanding, with measurements constrained by calibration complexity, strict sample-handling requirements, and limited standardization across studies [8,9]. These practical and methodological challenges make large-scale, systematic measurement campaigns difficult to implement, even with modern instrumentation [8], resulting in poor ground-truth datasets, as data acquisition processes are time-consuming, resource-intensive, and difficult to scale across diverse physiological conditions.

As a direct consequence of these constraints, the available dataset in this work is limited to a discrete set of Hb concentrations [10]. While each measurement provides detailed frequency-dependent spectral information, the dataset remains sparse regarding Hb levels. This imbalance, high spectral resolution but limited physiological coverage, restricts the ability to construct a general and comprehensive dataset for microwave-based Hb sensing. Ideally, a comprehensive dataset would span a wide and densely sampled range of Hb values; however, in practice, only discrete Hb levels can be measured, leading to incomplete coverage of the physiological range.

Data-driven models depend on sufficiently large and well-distributed datasets to learn stable relationships [11]. When data are sparse, models lack sufficient variability to learn stable relationships and become prone to overfitting, a limitation widely noted in machine learning research on sparse and unevenly distributed datasets [12], and may even exhibit degraded validation performance [13]. Complex-permittivity measurements of biological tissues are inevitably constrained to a small number of samples. This situation highlights the need for strong prior domain knowledge, as well as larger and more comprehensive datasets. One approach to addressing this limitation is the use of synthetic data. Synthetic data refers to artificially generated samples designed to replicate the statistical characteristics of real-world datasets [14]. Synthetic data can help reconstruct plausible spectra for unmeasured Hb values and improve the coverage of otherwise sparse measurements. However, widely used synthetic-data-generation methods, such as generative adversarial networks (GANs) and variational autoencoders (VAEs), are often ineffective in low-data settings, as they typically require large and diverse training datasets to learn reliable distributions [15]. This makes them unsuitable for the extremely small-sample conditions characteristic of biomedical microwave measurements. This gap motivates our work, which focuses on synthetic spectrum generation tailored to these constraints.

Further motivation arises from the well-established principle that machine-learning models perform more reliably when trained on larger, more diverse datasets. Small datasets promote overfitting, whereas increasing data volume systematically mitigates it [16]. Prior studies demonstrate that these benefits extend to classical classifiers, with synthetic data augmentation techniques improving the performance of models such as Naive Bayes in imbalanced data scenarios [17]. Moreover, synthetic data has been shown to significantly improve classification performance and minority class detection in an imbalanced tabular dataset [18]. Collectively, these studies motivate the development of robust data synthesis methods for permittivity-based blood analysis, along with dedicated machine-learning models capable of directly predicting blood analyte concentrations (e.g., hemoglobin (Hb), hematocrit (Hct), and glucose) from permittivity data. Based on the existing literature [19,20], interpolation and regression techniques provide a principled means of reconstructing the underlying functional relationship between sparsely sampled data points, particularly when only limited observations are available, and the continuous form of the function must be estimated rather than directly measured [21]. In such settings, Gaussian Process Regression (GPR) is especially effective, as it infers a smooth, continuous function with associated uncertainty estimates, enabling the intervening space between observations to be modelled coherently [22].

Building on the limitations of sparse biomedical datasets and the suitability of interpolation and probabilistic methods for synthetic data generation discussed in the literature, this work adopts structured data-synthesis strategies to address the sparsity of permittivity-based hemoglobin (Hb) measurements. By exploiting the functional relationship between Hb concentration and complex permittivity, we perform Hb-dependent filling, generating data at previously unmeasured Hb levels together with their corresponding frequency-dependent permittivity spectra. This process leverages interpolation and probabilistic methods, such as Gaussian Process Regression, to ensure physically consistent reconstruction. As a result, the initially discrete dataset, consisting of seven Hb levels, is densified into a densely sampled and physiologically consistent Hb range while preserving spectral behavior. The resulting synthetic dataset is validated and used to train predictive models for Hb estimation from microwave measurements. Our contributions are threefold:

- We propose a structured data-synthesis framework that performs Hb-dependent filling by generating new Hb levels together with their corresponding frequency-dependent permittivity spectra, enabling densification of sparse experimental data into a densely sampled Hb range.
- We develop a regression framework for predicting blood analyte concentrations from complex permittivity spectra, with hemoglobin (Hb) used as a representative target.
- We demonstrate that the densified dataset improves prediction accuracy and generalization across different Hb levels compared to models trained on sparse measurements alone.

## 2. Methods

**Figure 1:**
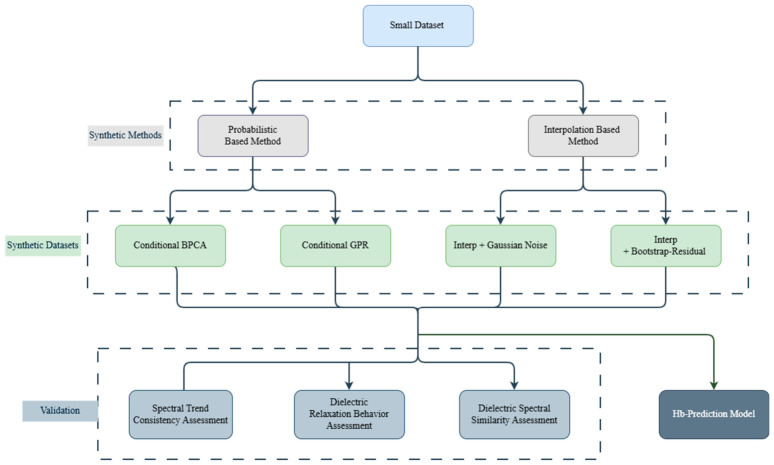
Overview of the proposed work.

This study, as illustrated in Figure 1, begins by generating synthetic permittivity spectra across a densely sampled range of hemoglobin values using two method families: interpolation-based techniques and probabilistic generative models. For each donor-specific dataset and each generation method, the synthetic Hb grid consisted of 1000 evenly spaced Hb levels between the minimum and maximum measured Hb concentrations for that donor. For each generated Hb level, two corresponding permittivity spectra were produced, one for $\varepsilon^{\prime }$ and one for $\varepsilon^{\prime \prime }$, with each spectrum sampled at 861 frequency points. Each generated dataset was evaluated in terms of agreement with measured trends, physical consistency based on the Cole–Cole relaxation model [23], distributional similarity to measured permittivity spectra using Earth Mover’s Distance [24], and variance preservation between measured and synthetic spectra. Finally, a tuned XGBoost regression model was trained on each dataset to assess Hb prediction performance and compare the effectiveness of the different synthesis strategies. All data processing, modeling, and analysis were performed using MATLAB (MathWorks, Natick, MA, USA, version R2024a) and Python (versions 3.12.3 and 3.10.19).

### 2.1. Measured Dataset

The dataset used in this study originates from previously prepared blood samples described in [10]. The dataset was prepared using 20 donated red blood cell (RBCs) preserved in saline–adenine–glucose–mannitol (SAGM) solution, which serves as the storage and suspending medium to preserve the life of the RBCs. From these donations, seven target hematocrit (Hct) levels were generated, covering a broad range of RBCs concentrations, i.e., 0%, 26%, 36%, 46%, 56%, 66%, plus a sample of highly concentrated RBCs with an equivalent Hct higher than 90%. With these levels, we covered all scenarios, from no RBCs (Hb = 0) to physiologically relevant hematocrit levels and packed RBCs samples.

To prepare the different hematocrit levels, each donated blood sample was first centrifuged to separate the RBCs from the surrounding liquid components. Following centrifugation, The RBCs fraction was recombined with the SAGM suspending medium in predefined ratios to generate the target hematocrit levels. Manual pipetting was used to control the volume of RBCs and suspending medium added to each sample, allowing for the desired hematocrit concentration to be constructed with consistency across the dataset.

For the 0% Hct condition, only the suspending medium was used, representing a sample without RBCs. For the intermediate hematocrit levels, increasing volumes of RBCs were mixed with decreasing volumes of SAGM to obtain the target concentrations of 26%, 36%, 46%, 56%, and 66%. The greater than 90% Hct condition was produced using a highly concentrated RBCs fraction, representing a packed-cell sample with minimal suspending medium. This preparation approach enabled the creation of a controlled dataset spanning low, normal, high, and extremely high hematocrit conditions.

Hemoglobin (Hb) concentration, measured in g/dL using a RAPID Point 500e blood gas analyzer (Siemens Healthineers, Erlangen, Germany), is closely associated with hematocrit (Hct), as both parameters reflect the underlying RBCs mass; therefore, higher RBC content generally corresponds to higher Hb [5]. Thus, each reconstructed Hct level yielded a distinct Hb measurement. Small variations introduced by manual pipetting resulted in differences among the seven subsamples within each donor; this preparation method produced a set of Hb values for each participant spanning low to high concentrations, consistent with the reconstructed Hct levels, which enriched the dataset with a reliable number of unique Hb values.

For each sample, real and imaginary components of the complex permittivity, $\varepsilon^{\prime }$ and $\varepsilon^{\prime \prime },$ were measured using a vector network analyzer (N5224A PNA, Keysight Technologies, Santa Rosa, CA, USA) and a coaxial probe over the frequency range of 0.5–43.5 GHz with 861 measurement points. Complex permittivity is expressed as $\varepsilon^{*}=\varepsilon^{\prime }-j\varepsilon^{\prime \prime }$, where $\varepsilon^{\prime }$ is the real part, corresponding to the dielectric constant, and $\varepsilon^{\prime \prime }$ is the imaginary part, corresponding to the dielectric loss factor [25]. A proper calibration procedure was followed and uncertainty analysis was performed [10].

Importantly, synthetic data generation was carried out independently for each donor, using only that donor’s own subsampled blood measurements rather than pooling data across donors. This was done to preserve donor-specific spectral structure and to ensure that the generated variations remained physiologically plausible, so that the observed changes primarily reflect Hb-dependent variation within a donor rather than differences between donors.

### 2.2. Synthesis Methods

#### 2.2.1. Interpolation-Based Methods

Interpolation-based methods generate continuous estimates from discrete measurements by constructing functions that preserve the underlying trends of the data. In this work, such a method is used to model the dependence of permittivity on hemoglobin concentration (Hb) and to generate synthetic spectra from a limited set of measured points. This approach is chosen because the available dataset contains only a few Hb levels, requiring a method that can produce reliable intermediate values without introducing non-physical behavior.

To achieve this, a shape-preserving interpolation framework based on the Piecewise Cubic Hermite Interpolating Polynomial is applied [26], because it maintains the monotonicity and qualitative structure of the measured data, avoiding the artificial oscillations that can arise with standard cubic spline interpolation, particularly in sparsely sampled datasets.

For each frequency, the real and imaginary components of permittivity are interpolated across Hb using PCHIP to produce smooth and physically consistent trends. The deviations between these interpolated trends and the measured data are then quantified as residuals, which form the basis for constructing two distinct synthetic datasets.

- Gaussian-Noise Synthetic Dataset
  Residual variability is represented using a Gaussian model. Synthetic values were obtained by adding normally distributed perturbations, scaled by the empirical residual standard deviation at each frequency, to the PCHIP-interpolated trend. This produced smooth synthetic spectra with controlled, symmetric variability.
- Bootstrap-Residual Synthetic Dataset
  Residual variability is captured non-parametrically using bootstrap resampling. Residuals are drawn with replacement from the observed residual set and added to the PCHIP trend. This preserved the empirical residual distribution, including potential asymmetry or heavy tails.

All synthetic values were restricted to the physically observed range for each frequency to prevent unrealistic excursions. These two variants, therefore, offered complementary interpolation-based approaches: one assumed Gaussian-like variability, and one preserved the empirical distribution of deviations.

#### 2.2.2. Probabilistic-Based Methods

Probabilistic-Based Methods model the relationship between hemoglobin concentration and permittivity spectra within a probabilistic framework. Instead of producing a deterministic estimate, these models learn a distribution over possible spectra conditioned on the observed measurements. This allows synthetic spectra to be generated while explicitly accounting for uncertainty in regions where experimental data are sparse. Two probabilistic modelling frameworks were developed:

- Conditional Gaussian Process Regressor

**Figure 2:**
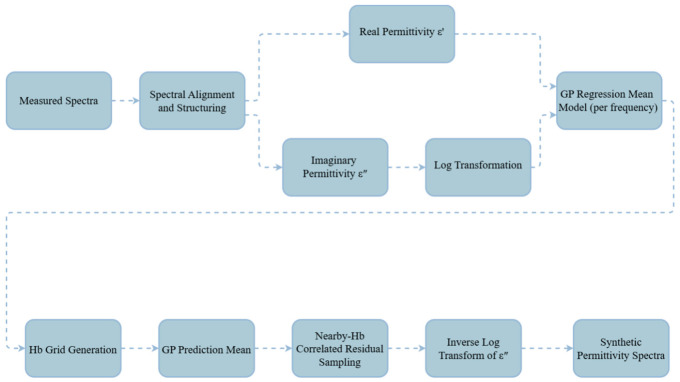
Workflow of the conditional Gaussian process framework for generating synthetic permittivity spectra.

While the previous approaches rely on interpolation-based techniques, this method adopts a probabilistic modelling approach based on Gaussian Process Regression (GPR) to generate synthetic permittivity spectra conditioned on hemoglobin concentration (Hb). A Gaussian Process defines a distribution over functions and is fully specified by a mean function and a covariance kernel, which determines the correlation and smoothness properties of the relationship between variables [27]. The overall workflow of the conditioned Gaussian Process framework is illustrated in Figure 2.

In this approach, the permittivity values at each frequency index are modelled as functions of Hb across the set of measured samples. Accordingly, a Gaussian Process is fitted independently at each frequency point to capture how the real and imaginary components of permittivity vary with Hb concentration.

For each frequency index j, the functional relationship is represented as a Gaussian Process prior [27]

(1) $$f_{j}(Hb)∼GP(m_{j}(Hb),\;\;k_{j}(Hb,{Hb}^{\prime }))$$

where $m_{j}(Hb)$ is the mean function and $k_{j}(Hb,{Hb}^{\prime })$ is the covariance kernel. In this work, the covariance function was defined using a constant kernel multiplied by a radial basis function (RBF) kernel, with an additional white-noise kernel to account for observation noise. The same kernel structure was used across all frequency-specific GPR models, while the optimized hyperparameters included the signal variance, RBF length scale, and white-noise level. These hyperparameters were optimized separately for each frequency-specific model.

After conditioning on the observed *Hb* measurements, the Gaussian Process provides a predictive Gaussian distribution for any new *Hb* value, Hb*, as expressed in Equation (2)

(2) $$f_{j}({Hb}^{*})|D∼N(\mu_{j}({Hb}^{*}),\;\sigma_{j}^{2}({Hb}^{*}))$$

where $\mu_{j}({Hb}^{*})$ and $\sigma_{j}^{2}({Hb}^{*})$ are the predictive mean and variance of the Gaussian process prediction, respectively, following standard GP regression [27].

The imaginary component of permittivity is modelled in the log-transformed domain, expressed as

(3) $$y_{\mathrm{imag}}=log(max(\varepsilon^{\prime \prime },0)+\epsilon )$$

where ϵ is a small constant added for numerical stability. This transformation stabilizes the variance and improves the smoothness of the Gaussian Process fit, as the imaginary component often varies multiplicatively with concentration and frequency. Modelling in the log domain also ensures that generated values remain physically meaningful by enforcing non-negativity after the following inverse transformation.

(4) $$\varepsilon^{\prime \prime }=exp(y_{\mathrm{imag}})-\epsilon$$

Synthetic spectra are generated by evaluating the Gaussian Process predictive distributions on a dense Hb grid spanning the range of the measured concentrations. For a given Hb value, the model predicts the permittivity at each frequency index, and these predicted values are combined to reconstruct the full spectrum.

The final Conditional GPR implementation was designed to avoid independent frequency-point perturbations. Although the GPR mean models were fitted separately at each frequency point, variability was introduced using correlated full-spectrum residual patterns sampled from nearby Hb values. This allowed the generated spectra to preserve smoother frequency-dependent structure while retaining measured-data-derived variability.

After sampling, the imaginary component is mapped back from the logarithmic domain using the inverse exponential transformation and clipped at zero to maintain non-negativity. The resulting synthetic spectra follow smooth nonlinear Hb-dependent trends while incorporating correlated residual variability derived from the measured spectra.

II. Conditional Bayesian Principal Component Analysis

**Figure 3:**
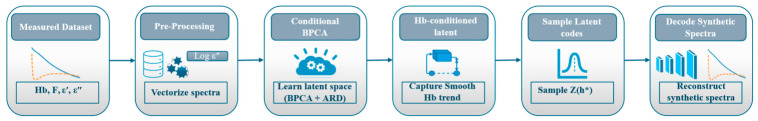
Workflow of the Conditional BPCA framework for generating synthetic permittivity spectra.

Conditional Bayesian Principal Component Analysis (CBPCA) is based on the idea that high-dimensional permittivity spectra can be represented by a small number of latent factors whose distribution varies with an underlying conditioning variable [28]. The overall workflow of the CBPCA framework used for synthetic spectrum generation is illustrated in Figure 3.

This approach is based on a probabilistic PCA formulation [28] with Automatic Relevance Determination (ARD)-style shrinkage [29], in which each vectorized spectrum is represented by

(5) $$x=\mu_{x}+Wz+\epsilon$$

where x is the vectorized spectrum formed by concatenating the real and imaginary components, $\mu_{x}$ is the mean spectrum, W is the loading matrix, z is a latent variable, and ϵ represents isotropic noise. The maximum number of latent components was set to 64. The final retained latent dimensionality was selected automatically using ARD-based pruning, where components with negligible contribution were removed after model fitting. Therefore, the retained number of components was data-dependent for each donor-specific model.

The imaginary component is transformed using logarithmic mapping after non-negative flooring, as

(6) $$y_{\mathrm{imag}}=log(1+max(\varepsilon^{\prime \prime },0))$$

which stabilizes the scale of the response and improves numerical behavior. This transformation also ensures that generated imaginary values can be mapped back to a non-negative domain after sampling.

To incorporate physiological variation, the latent structure is conditioned on hemoglobin concentration. Let h denote the normalized Hb value, defined as

(7) $$h=\frac{Hb-\mu_{Hb}}{\sigma_{Hb}}$$

where $\mu_{Hb}$ and $\sigma_{Hb}$ denote the mean and standard deviation of the observed Hb values, respectively. The conditional latent mean is modelled as a nonlinear function of h, as represented in the following formula

(8) $$\mu_{z}(h)=b_{0}+b_{1}h+\overset{M}{\underset{m=1}{\sum }}c_{m}\;\phi_{m}(h)$$

where $\phi_{m}(h)$ are radial basis functions, and $b_{0}$, $b_{1}$, and $c_{m}$ are coefficient vectors in the latent space.

To capture concentration-dependent variability, residual latent covariance matrices are estimated within discrete Hb bins. For a new normalized concentration h*, the covariance is selected from the nearest bin. If a bin contains insufficient samples, a global residual covariance estimated across all Hb values is used as a fallback.

Synthetic spectra are generated by sampling from the following conditional latent distribution.

(9) $$z(h^{*})∼N(\mu_{z}(h^{*}),\Sigma_{z}(h^{*}))$$

where $\Sigma_{z}(h^{*})$ is the residual latent covariance associated with the nearest *Hb* bin. The sampled latent vector is then mapped back to the spectral domain through

(10) $$x_{\mathrm{syn}}(h^{*})=\mu_{x}+Wz(h^{*})$$

Finally, the imaginary component is returned to its original domain using the inverse transformation as

(11) $$\epsilon^{\prime \prime }=exp(y_{\mathrm{imag}})-1$$

followed by non-negativity clipping. In this way, the CBPCA framework generates synthetic spectra that capture global spectral structure, nonlinear Hb-dependent trends, and concentration-dependent variability.

### 2.3. Validation Approaches

This section describes the validation approaches used to assess how closely the synthetic dielectric spectra replicate the characteristics of the measured data. Multiple complementary methods are applied to capture visual, physical, statistical, and predictive alignment.

#### 2.3.1. Spectral Trend Consistency Assessment

The process begins with a qualitative assessment of spectral consistency by overlaying measured and synthetic dielectric curves across the frequency range. Each measured Hb value is paired with its closest synthetic counterpart, while enforcing a minimum spacing of 0.5 Hb units to ensure visual distinguishability between curves. The real and imaginary components of the dielectric response for each pair are plotted using the same color with different line styles, enabling direct visual comparison and facilitating the evaluation of how closely the synthetic data follows the trends observed in the measured spectra.

#### 2.3.2. Variance Preservation Assessment

To quantitatively assess potential over-smoothing, a variance preservation analysis [30] was performed across all donor-specific datasets. For each donor-specific dataset and each synthetic generation method, measured and synthetic spectra were arranged into frequency-aligned matrices, with rows representing spectra and columns representing frequency points. The frequency-wise variance was then calculated separately for ε′ and ε″.

For each donor-specific dataset, the variance across frequency was averaged for the measured spectra and for the corresponding synthetic spectra. Variance preservation was summarized using the synthetic-to-measured variance ratio:

(12) $$Variance\;Ratio=\frac{Mean\;synthetic\;variance\;across\;frequency}{Mean\;measured\;variance\;across\;frequency}$$

A ratio close to one indicates that the synthetic spectra preserve a similar level of variability to the measured spectra. Ratios substantially below one indicate reduced variability and possible over-smoothing, whereas ratios above one indicate greater variability than the measured data. The variance ratio was calculated separately for $\varepsilon^{\prime }$ and $\varepsilon^{\prime \prime }$ within each donor-specific dataset. For each synthetic generation method, the donor-level variance ratios were then averaged across all donor-specific datasets. The error bars in the plot represent the standard deviation of these donor-level variance ratios across donors, indicating the consistency of variance preservation across datasets.

#### 2.3.3. Dielectric Relaxation Behavior Assessment

The dielectric response of both measured and synthetic spectra was parameterized using the Cole–Cole model, which is widely used as a standard representation of the frequency-dependent permittivity of biological tissues [31]. The Cole–Cole formulation has been extensively adopted in bio-electromagnetics due to its ability to capture the distributed nature of dielectric relaxation processes in heterogeneous biological media. In particular, the seminal work of Gabriel and co-authors [7] demonstrated that multi-pole Cole–Cole models can accurately represent the dielectric properties of a wide range of biological tissues over broad frequency bands, and this approach has since become a common parametric framework in the literature.

The complex permittivity was modelled using the Cole–Cole expression that is shown in Equation (13) [32]

(13) $$\varepsilon^{*}(\omega )=\varepsilon_{\infty }+\frac{\Delta \varepsilon_{1}}{1+(j\omega \tau_{1}{)}^{1-\alpha_{1}}}+\;\frac{\Delta \varepsilon_{2}}{1+(j\omega \tau_{2}{)}^{1-\alpha_{2}}}$$

where $\varepsilon_{\infty }$ is the permittivity at the high-frequency limit, $\Delta \varepsilon_{1}$ and $\Delta \varepsilon_{2}$ are the relaxation strengths associated with the respective dispersion processes, $\tau_{1}$ and $\tau_{2}$ are the corresponding relaxation times, and $\alpha_{1}$ and $\alpha_{2}$ are the broadening parameters satisfying $0<\alpha_{i}<1$, accounting for the distribution of relaxation times. Here, ω=2πf denotes the angular frequency.

A second-order Cole–Cole model is employed to capture multiple relaxation contributions that cannot be adequately described by a single term, particularly in the GHz frequency range where the dominant water-related relaxation may include contributions from bound water and macromolecular interactions.

The conductivity term is neglected, as its contribution is negligible above 0.5 GHz compared to dielectric polarization. Higher-order models are avoided to prevent over-parameterization, which can lead to non-unique parameter estimation, where different parameter sets yield similarly accurate fits and reduce physical interpretability.

For each spectrum, the complex permittivity was fitted across the frequency range used for comparison to obtain a smooth Cole–Cole representation. Before fitting, the frequency values were converted to angular frequency and normalized by the maximum angular frequency to improve numerical stability. The real and imaginary components of the complex permittivity were fitted jointly by concatenating both components into a single nonlinear least-squares objective. Model parameters were estimated using bounded nonlinear least-squares optimization with fixed initialization and predefined parameter bounds. The fitted parameter vector was ordered as ([$\varepsilon_{\infty }$, $\Delta \varepsilon_{1}$, $\tau_{1}$, $\alpha_{1}$, $\Delta \varepsilon_{1}$, $\tau_{2}$, $\alpha_{2}$]). The initial parameter values were set to ([4, 40, $10^{-1}$, 0.2, 20, 1.0, 0.2]). The lower bounds were ([0, 0, $10^{-6}$, 0, 0, $10^{-6}$, 0]), and the upper bounds were ([100, 300, $10^{3}$, 1, 300, $10^{3}$, 1]). Because the angular frequency was normalized before fitting, $\tau_{1}$ and $\tau_{2}$ were optimized in the normalized angular-frequency domain. The maximum number of function evaluations was set to 200,000. Fits that failed to converge or produced invalid numerical outputs were excluded from the Cole–Cole matching procedure. The quality of each fit was assessed by computing the root mean square error (RMSE) between the raw and fitted complex permittivity. This step was used to verify that the Cole–Cole model provided an adequate smooth representation of the dielectric response.

Next, the similarity between measured and synthetic samples was evaluated using their fitted complex permittivity spectra in the measured-to-synthetic direction. For each measured Hb spectrum, the fitted Cole–Cole response was compared with the fitted responses of all eligible synthetic candidates from the corresponding donor-specific synthetic dataset. The discrepancy between the two spectra was quantified using the RMSE defined directly on the complex permittivity, as shown in Equation (14):

(14) $$RMSE=\sqrt{\frac{1}{N}\overset{N}{\underset{i=1}{\sum }}{|\varepsilon_{meas,fit}(f_{i})-\varepsilon_{syn,fit}(f_{i})|}^{2}}$$

where $\varepsilon_{meas,fit}(f_{i})$ and $\varepsilon_{syn,fit}(f_{i})$ denote the fitted complex permittivity values of the measured and synthetic spectra at frequency $f_{i}$, and N is the number of frequency points. The synthetic spectrum yielding the minimum RMSE was identified as the closest match to the corresponding measured spectrum. To reduce potential bias from direct overlap, synthetic samples with identical Hb values to the measured spectrum were excluded from the candidate set during matching. This procedure was repeated for all corresponding donor-specific measured and synthetic datasets.

#### 2.3.4. Dielectric Spectral Similarity Assessment

Following the model-based comparison using the Cole–Cole formulation, similarity between measured and synthetic dielectric spectra was further evaluated directly from the raw spectral data using the Earth Mover’s Distance (EMD). In this framework, each dielectric spectrum is interpreted as a distribution of mass along the frequency axis, and the EMD quantifies the minimum cost required to transform one distribution into another.

EMD is mathematically equivalent to the first-order Wasserstein distance [24], and is defined as shown in Equation (15):

(15) $$W_{1}(P,Q)=\underset{\gamma \in \Pi (P,Q)}{inf}\int |x-y|\;d\gamma (x,y)$$

where P and Q denote the two distributions being compared, x and y represent positions along the frequency axis, and Π(P,Q) is the set of admissible transport plans between the two distributions. The quantity ∣x−y∣ represents the distance that mass must be transported along the frequency axis, and the integral corresponds to the total transport cost required to transform one spectral distribution into the other.

For each spectrum, the real $\varepsilon^{\prime }$ and imaginary $\varepsilon^{\prime \prime }$ components were restricted to their common frequency-overlap region. Each component was then converted into a discrete mass distribution by weighting the dielectric response by the local frequency spacing Δf expressed in Equation (16)

(16) $$m_{i}=\left|\varepsilon_{i}\right|\;\Delta f_{i}$$

The resulting masses were normalized to form discrete probability distributions suitable for EMD computation. Thus, EMD was used to assess the relative frequency-dependent distribution of the dielectric response rather than absolute amplitude alone; amplitude information was retained through the relative magnitude of the real and imaginary components across frequency before normalization.

One-dimensional EMD values were computed separately for the real and imaginary components and combined into a single similarity measure using a root-mean-square formulation expressed in Equation (17)

(17) $$EMD_{\mathrm{combined}}=\sqrt{\frac{1}{2}\left(EMD_{\varepsilon^{\prime }}^{2}+EMD_{\varepsilon^{\prime \prime }}^{2}\right)}\;$$

Because synthetic data generation was performed independently for each donor’s measured data, evaluation was likewise restricted to donor-matched measured and synthetic subsets to prevent cross-donor leakage and overly optimistic similarity estimates. For evaluation, each measured Hb spectrum from a given donor was compared only with the corresponding synthetic dataset generated for that same donor. Within each donor-specific dataset, every measured Hb spectrum was compared against all eligible synthetic spectra for a given generation method, and the minimum combined EMD was retained. To reduce potential bias from direct overlap, synthetic samples with identical Hb values to the measured spectrum were excluded from the candidate set. This identifies, for each measured case, the closest synthetic approximation in terms of spectral distribution. To compare the four generation methods across all donors’ datasets, the retained minimum EMD values were summarized at the donor level. Specifically, for each method and donor, the mean of the retained minimum Measured-to-Synthetic combined EMD values was calculated, yielding one summary score per donor per method. Boxplot-based visualizations were then constructed from these donor-level summary scores rather than from all pooled pairwise EMD values. This donor-level aggregation provides a consistent basis for comparing the overall agreement and variability of the four synthetic generation methods across donors. Lower EMD values indicate better agreement between measured and synthetic spectra.

#### 2.3.5. Predictive Behavior Assessment

To assess the predictive utility of the synthetic spectral datasets, an XGBoost regression model was trained to infer Hb concentration from synthetic complex permittivity spectra and evaluated on held-out measured spectra. XGBoost was used as the regression engine due to its ability to capture nonlinear patterns while maintaining strong regularization [33]. Before modelling, each complex permittivity curve was expressed in polar form, using magnitude and phase across the full frequency range, providing a structured representation of both dielectric strength and dispersive behavior. These features were then arranged into a consistent wide format and standardized using z-scores to ensure uniform scaling across spectra.

To avoid overly optimistic evaluation due to correlations between spectra derived from the same measured source, a grouped cross-validation strategy was adopted. The evaluation was organized into four donor-group folds. In each fold, the model was trained and tuned using only synthetic spectra corresponding to three donor groups and was externally evaluated on the measured spectra from the held-out donor group. The held-out measured donor group, and any synthetic spectra corresponding to that group, were not used during model training, hyperparameter tuning, preprocessing, standardization, or feature scaling.

Hyperparameter optimization was performed using Optuna’s Tree-structured Parzen Estimator (TPE) [34]. For each training fold, the objective was to minimize the mean RMSE of hemoglobin (Hb) prediction, estimated via an internal Group K Fold procedure applied to the synthetic training data. The search space included key XGBoost parameters such as learning rate, tree depth, child weight, subsampling ratios, feature sampling, regularization terms (gamma, L1, L2), and the number of boosting rounds.

The final model for each fold was trained on all synthetic spectra from the selected training donor groups and evaluated only on the corresponding held-out measured permittivity spectra. The measured data were pre-processed consistently with the synthetic data, including conversion to magnitude and phase, frequency alignment, and standardization using training-set statistics.

**Figure 4:**
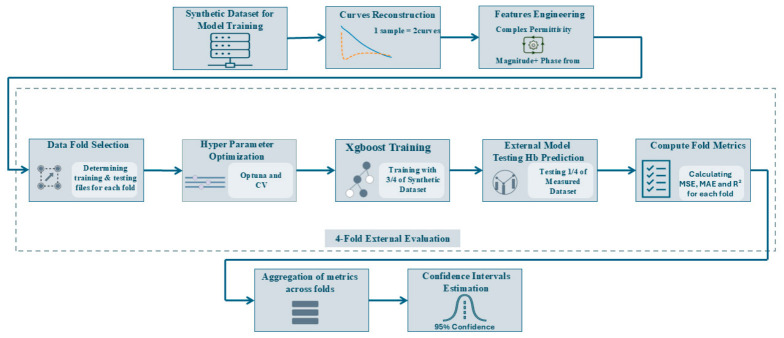
Block diagram of the proposed Hb estimation model.

Model predictions were compared to measured Hb values using multiple metrics, including RMSE, MAE, and R^2^. This evaluation provided a direct measure of how effectively a model trained solely on synthesized spectra could generalize to real experimental measurements. To quantify uncertainty in the evaluation, 95% confidence intervals were estimated using fold-level percentile bootstrap resampling with 5000 resamples [35]. After completion of the four donor-group cross-validation folds, the held-out donor-group folds were resampled with replacement as whole units to preserve the grouped structure of the external validation design. For each bootstrap replicate, the actual–predicted pairs were pooled across the resampled folds, and RMSE, MAE, and R^2^ were recomputed. The 2.5th and 97.5th percentiles of the resulting bootstrap distributions were used as the 95% CI bounds. Figure 4 illustrates the workflow.

## 3. Results

### 3.1. Spectral Trend Consistency Assessment Results

**Figure 5:**
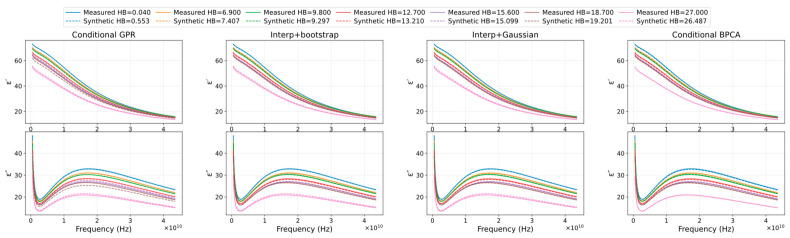
Comparison of measured and synthetic permittivity spectra across frequency and Hb levels for each synthesis method.

The plots in Figure 5 show that all methods capture the characteristic dielectric behavior associated with the Hb response. Although small differences are observed between methods, the overall trend remains consistent across them. The curves shown represent one donor subsample, while the synthetic datasets generated for the other donors exhibit the same general trend.

### 3.2. Variance Preservation Assessment Results

**Figure 6:**
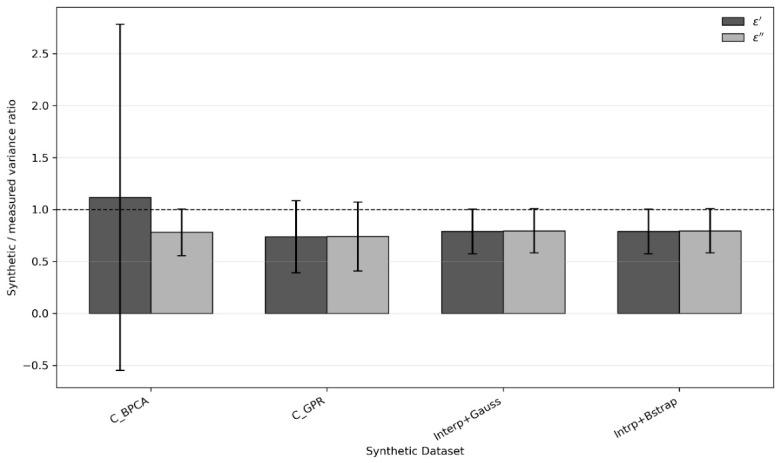
Comparison of measured and synthetic variance across synthesis methods.

Figure 6 assesses variance preservation across donor-specific datasets via the synthetic-to-measured variance ratio for ε′ and ε″, where values near unity indicate retained variability. Most ratios fell slightly below one, reflecting moderate smoothing without severe variance collapse. Interpolation-based methods and Conditional GPR showed consistent preservation across donors, whereas Conditional BPCA exhibited greater donor-dependent variability, particularly for ε′. In general, the synthetic spectra retained meaningful spectral variability rather than converging toward overly smooth average curves.

### 3.3. Dielectric Relaxation Behavior Assessment Results

**Figure 7:**
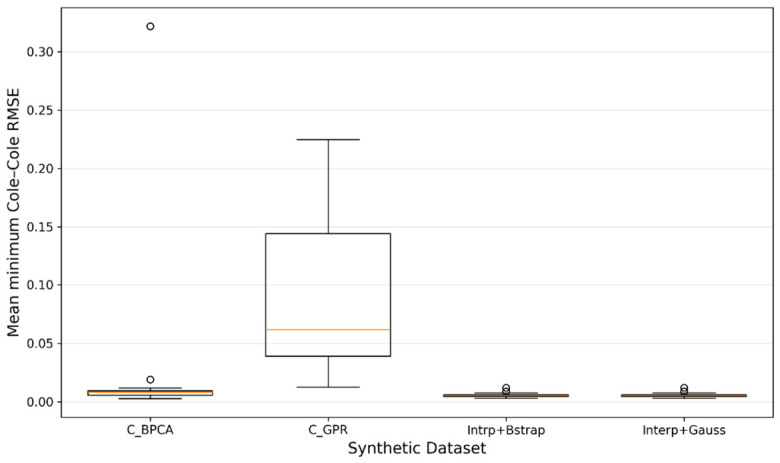
Mean minimum Cole–Cole RMSE between measured and synthetic permittivity spectra across synthesis methods.

Figure 7 shows that the interpolation-based methods achieve the lowest mean minimum fit-to-fit RMSE values, with near-zero medians and narrow spreads, indicating strong agreement between fitted measured and synthetic spectra. Conditional BPCA also performs well, although one outlier suggests reduced consistency for a single donor dataset. In contrast, Conditional GPR shows the highest RMSE values and widest spread, indicating greater variability and larger discrepancies between fitted spectra.

### 3.4. Dielectric Spectral Similarity Assessment Results

**Figure 8:**
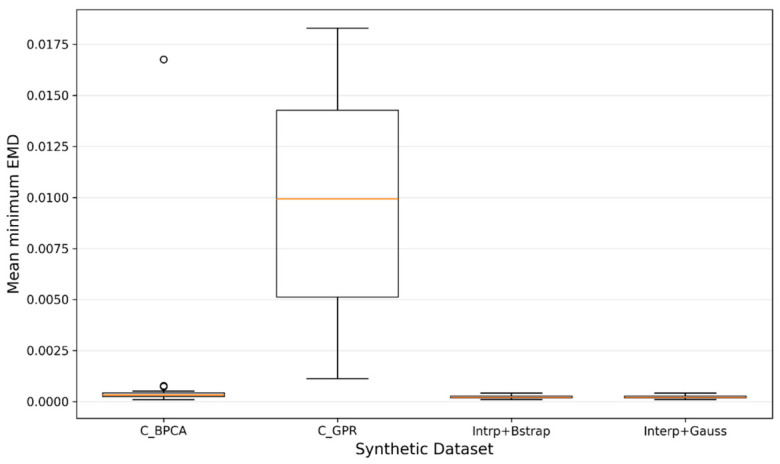
Mean minimum combined Earth mover’s distance between measured and synthetic dielectric spectra across synthesis methods.

Figure 8 shows that the interpolation-based methods provide the lowest mean minimum EMD values in the measured-to-synthetic direction, with near-zero medians and narrow spreads, indicating close agreement between measured and synthetic spectra. Conditional BPCA shows slightly higher but generally well-clustered EMD values, with one outlier suggesting reduced consistency for a single donor dataset. On the other hand, Conditional GPR has the highest median and broadest spread, indicating less consistent agreement with the measured spectra, although its overall EMD values remain relatively low.

### 3.5. Predictive Behavior Assessment Results

Table 1 summarizes Hb estimation performance across the measured-only baseline and the synthetic datasets using point estimates with fold-level bootstrap 95% confidence intervals for RMSE, MAE, and R^2^. Overall, the synthetic datasets showed broadly comparable predictive performance to the measured-only baseline.

Among the synthetic datasets, the interpolation-based methods produced the lowest RMSE and highest R^2^ values. Interpolation + Gaussian Noise achieved RMSE = 1.684 [1.347, 1.998], MAE = 1.248 [1.100, 1.416], and R^2^ = 0.949 [0.922, 0.969]. Interpolation + Bootstrap Residual showed very similar performance, with RMSE = 1.696 [1.347, 2.020], MAE = 1.253 [1.100, 1.428], and R^2^ = 0.948 [0.920, 0.969].

Conditioned BPCA also showed comparable performance, with RMSE = 1.774 [1.447, 1.997], MAE = 1.337 [1.129, 1.517], and R^2^ = 0.943 [0.917, 0.966]. Conditioned GPR showed slightly higher prediction error, with RMSE = 1.948 [1.500, 2.500], MAE = 1.403 [1.148, 1.754], and R^2^ = 0.932 [0.870, 0.963]. However, the confidence intervals overlapped with those of the other datasets, indicating broadly comparable predictive performance.

These results indicate that the synthetic spectra retained Hb-related information useful for downstream regression, with the interpolation-based methods showing the strongest predictive performance among the evaluated synthetic datasets.

To statistically compare predictive performance among the four synthetic generation methods, fold-level RMSE, MAE, and R^2^ values were evaluated using the Friedman test [36]. The Friedman test is a non-parametric test used to compare multiple related methods and was selected because the same held-out donor groups were evaluated across all synthetic generation methods. No statistically significant differences were observed among the measured and synthetic datasets for RMSE, MAE, or R^2^, as all *p*-values were above 0.05, indicating comparable predictive performance under the XGBoost evaluation framework.

## 4. Discussion

The findings of this study reveal a nuanced landscape in which different synthetic-data generation methods emphasise distinct aspects of spectral quality and predictive utility. A clear pattern emerges: approaches grounded in interpolation most closely follow the measured dielectric behaviour, while probabilistic models introduce flexibility at the cost of strict reconstruction fidelity.

Interpolation-based methods emerged as the most faithful to the measured spectra, consistently demonstrating the closest agreement across all evaluation metrics. Their strength lies in their ability to preserve the local structure of the original measurements, effectively anchoring the generated spectra within the observed donor-specific hemoglobin (Hb) range. This inherent constraint minimises reconstruction error, as evidenced by low Cole–Cole fit-to-fit RMSE and tightly clustered EMD distributions.

The variance preservation analysis reinforces this interpretation, showing that these methods retain a substantial proportion of the original spectral variability without collapsing into overly smoothed or simplified representations. In doing so, they maintain not only accuracy but also the natural heterogeneity present in the measured data.

Rather than attempting to reinterpret or generalise the underlying relationships, interpolation-based approaches remain closely tied to the observed data. This fidelity to measurement ultimately translates into strong and reliable predictive performance, highlighting their effectiveness when reconstruction accuracy is the primary objective.

However, the remaining discrepancies between measured and synthetic spectra suggest that Hb concentration alone does not fully explain the dielectric response of blood. Subtle yet influential factors, such as variations in water content, temperature fluctuations during measurement, and calibration uncertainty, introduce complexity into the spectral behaviour. Because microwave dielectric properties are highly sensitive to water-associated relaxation processes, even small shifts in the balance between RBCs and suspending medium can alter the observed spectra. These effects, though not explicitly modelled, contribute to reconstruction and prediction error across all methods.

Against this backdrop, Conditional BPCA presents a different narrative. Rather than adhering strictly to local measurements, it models the broader statistical structure underlying the data. This probabilistic perspective leads to smoother spectral outputs and slightly higher reconstruction error compared with interpolation. Yet it also brings notable stability. Across Cole–Cole analysis, EMD similarity, and variance preservation, Conditional BPCA maintains consistent behaviour, capturing the essential distribution of the data while preserving meaningful variability, particularly in the real component of permittivity. This balance highlights its strength as a generative framework.

Conditional GPR, in contrast, illustrates the challenges of learning from limited information. Its weaker reconstruction consistency and comparatively lower predictive accuracy suggest difficulty in capturing Hb-dependent spectral trends from sparse donor-specific inputs. Although its probabilistic nature allows for uncertainty quantification, the absence of additional explanatory variables appears to limit its effectiveness in this context.

Taken together, these observations emphasise that synthetic data quality cannot be reduced to a single definition. Interpolation methods excel in fidelity and prediction, BPCA achieves stability and realistic variability, and GPR offers a flexible but less precise modelling approach. The results underline the importance of evaluating synthetic data through multiple complementary lenses, including spectral agreement, physical plausibility, variance preservation, and predictive performance. Only by considering all of these dimensions can the strengths and limitations of each method be fully understood.

## 5. Conclusions

This study demonstrates that synthetic dielectric spectra can effectively reproduce key characteristics of measured blood permittivity and support haemoglobin (Hb) estimation under limited data conditions. Interpolation-based methods achieved the highest reconstruction fidelity and strongest predictive performance, while Conditional BPCA provided stable generative behaviour with good variability preservation. Conditional GPR showed comparatively weaker performance but still offered a probabilistic modelling perspective.

The results highlight that synthetic data quality must be assessed using multiple complementary criteria, including spectral fidelity, variance preservation, and predictive utility. Future work should incorporate additional variables, such as water content, temperature, and calibration uncertainty, to better capture underlying physical variability and improve both spectral generation and biomarker prediction.

More broadly, the proposed framework can be extended to other blood parameters beyond Hb, enabling the generation of richer synthetic datasets for multivariable analysis. This expansion has the potential to support improved prediction of multiple biomarkers, enhancing the applicability of microwave-based diagnostic models.

## Figures and Tables

**Table 1:** Bootstrap confidence intervals of the Hb estimation model across measured and synthetic datasets.

Data Sets	RMSE 95% CI	MAE 95% CI	R^2^ 95% CI
Measured	1.813 [1.157, 2.344]	1.169 [0.908, 1.465]	0.941 [0.893, 0.977]
Interpolation + Gaussian Noise	1.684 [1.347, 1.998]	1.248 [1.100, 1.416]	0.949 [0.922, 0.969]
Interpolation + Bootstrap Residual	1.696 [1.347, 2.020]	1.253 [1.100, 1.428]	0.948 [0.920, 0.969]
Conditioned BPCA	1.774 [1.447, 1.997]	1.337 [1.129, 1.517]	0.943 [0.917, 0.966]
Conditioned GPR	1.948 [1.500, 2.500]	1.403 [1.148, 1.754]	0.932 [0.870, 0.963]

## Data Availability

The datasets presented in this article are available from the authors.
